# Dynamic patterning of microparticles with acoustic impulse control

**DOI:** 10.1038/s41598-022-18554-5

**Published:** 2022-08-25

**Authors:** Luke Cox, Anthony Croxford, Bruce W. Drinkwater

**Affiliations:** Department of Mechanical Engineering, University of Bristol, University Walk, Bristol, BS8 1TR UK

**Keywords:** Mechanical engineering, Acoustics

## Abstract

This paper describes the use of impulse control of an acoustic field to create complex and precise particle patterns and then dynamically manipulate them. We first demonstrate that the motion of a particle in an acoustic field depends on the applied impulse and three distinct regimes can be identified. The high impulse regime is the well established mode where particles travel to the force minima of an applied continuous acoustic field. In contrast acoustic field switching in the low impulse regime results in a force field experienced by the particle equal to the time weighted average of the constituent force fields. We demonstrate via simulation and experiment that operating in the low impulse regime facilitates an intuitive and modular route to forming complex patterns of particles. The intermediate impulse regime is shown to enable more localised manipulation of particles. In addition to patterning, we demonstrate a set of impulse control tools to clear away undesired particles to further increase the contrast of the pattern against background. We combine these tools to create high contrast patterns as well as moving and re-configuring them. These techniques have applications in areas such as tissue engineering where they will enable complex, high fidelity cell patterns.

## Introduction

The ability to manipulate microscale objects is increasingly important in a range of fields, for example cell manipulation in bio-medicine. Optical tweezers allow high precision and the manipulation of individual particles/cells^1,2^, however due to the inefficient conversion from optical power to optical radiation force they are most commonly used for manipulation of a small number of particles rather than the creation of large scale multi-particle patterns. Magnetic fields can be useful for nanoscale alignment, however they either require extremely strong magnetic fields^3^ or labelling with magnetic particles^4^. Acoustic radiation force methods, typically operating at ultrasonic frequencies, have been used to produce engineered cell constructs which will enable more realistic in-vitro tissues for autologous grafting or fundamental research^5^. Acoustic radiation force approaches have also been used to pattern and fix polymer beads^6^, carbon fibres^7^ and hydrogels^8^. Acoustic manipulation offers unique capabilities in such applications; it is entirely label free, it can work on a large number of cells in parallel, it is potentially low cost and it does not adversely affect the viability of cells^9–11^. Many of these benefits stem from the efficiency of conversion from acoustic power to acoustic radiation force that is higher than in optical manipulation by the ratio of the speed of light to the speed of sound^12^. This makes acoustic manipulation possible with a few Watts of power.

Acoustic devices have been created that can manipulate individual particles^13,14^ or cells^15^ into the desired location one particle at a time. This has been demonstrated primarily using vortex traps which produce acoustic intensity distributions shaped as a first order Bessel function. Vortex traps have a high intensity ring that surrounds a low intensity core which traps a particle at the centre and by moving this ring the particle is moved. They can be generated either by a ring of sources in the plane of manipulation^16,17^ or projected from another plane using a beam^13^. This one-particle-at-a-time approach to patterning is a time consuming process and the particles have a minimum separation so that the forces on the particle under manipulation do not disturb the other particles.

Because high intensity acoustic fields can be formed over relatively large areas, a range of techniques have emerged in which a single continuous-wave pressure field is used to apply forces to many particles simultaneously and form them collectively into various patterns. The complexity of the pressure field created is related to the number of independently controlled ultrasound producing elements. As the number of elements increases so does the complexity of the achievable pattern^18^. One method of achieving complex acoustic field distributions is via a phased array, with electronically controlled elements producing a desired output phase and amplitude. For example, a U-shaped pattern was produced in simulation, although, as the force field contained features away from the U-shape, some particles were observed away from the desired shape^19^. Here, we note that particles that are trapped away from the desired shape or unpatterned can be thought of as artefacts or noise and act to lower the contrast of the pattern. Experimental results using arrays have been limited to regularly spaced lines and dots^19,20^. The complexity and cost of a phased array increases rapidly with the number of elements so larger arrays have not been explored. Alternatively, acoustic holograms have been produced using a 3D printed surface to apply static phase delays^21^. This approach allowed a complex line drawing of a dove of peace to be created, although many particles remained untrapped. Furthermore, the pattern achievable is fixed by the physical shape of the hologram. Field-programmable acoustic arrays are one approach to resolving this problem, using trapped gasses to stop the transmission of sound. However, while technically re-programmable this approach requires an extensive reset procedure to change the field^22^. Holographic microbubble arrays are a more easily changed approach to the same problem where an array of electrodes^23^ or a light projection^24^ generates bubbles across a plane wherever it is desired to block the sound. Such devices are faster to reset and have generated patterns shaped as letters which can be re-configured. However, they have yet to demonstrate high contrast particle patterning or dynamic manipulation of particle patterns.

Acoustic manipulation can also be conducted in air, where it is more commonly referred to as acoustic levitation. Multiple particles have been patterned and moved in parallel^25^ or independently^26,27^. As well as vortex traps, in-air levitation devices have utilised a twin trap where the particle is held between two regions of high pressure^26^. The twin trap is particularly simple to generate, requiring only two out-of-phase elements focused at a point^28^.

A number of researchers have explored the possibilities that emerge when acoustic fields are rapid modulated or switched between states. The simplest approaches involve applying short pulses; Takeuchi et al. showed short pulses of an ultrasonic travelling wave could move particles in the desired direction^29^. This idea has been applied in clinical settings for imaging purposes, acoustic radiation force impulse imaging involves using a short pulse of ultrasound to produce a deformation and the waves propagating from this are measured^30–32^. In the context of particle manipulation Collins et al.^33^ used sub-time-of-flight pulses to create localised areas of manipulation within a channel while Glynne-Jones et al.^34^ showed that by rapidly switching between the modes of a micro-channel with different trapping regions particles could be manipulated to any location across the micro-channel. More recently two vortex traps with opposite handed helicity were used to stably trap a large particle and control the rotation rate^35^. Switching rapidly between a pseudo-one-dimensional standing wave and a twin trap was also used to stably trap a non-spherical particle^36^.

In this paper we seek to generalise these field-switching manipulation concepts and describe an approach to patterning where the particle motion and location is determined by the impulse applied. This impulse is controlled either by the amplitude of a number of constituent pressure fields, or by the time they are applied to the particles. We then characterise the particle behaviour under three different impulse regimes: low, intermediate and high. By using the low impulse regime and switching rapidly between different pressure fields we demonstrate that a force field can be generated which is the time weighted sum of the force fields from the constituent pressure fields. This provides an intuitive and modular route to the production of a desired pattern of particles. Once the basic pattern is formed we then show that by using combinations of the impulse regimes we can induce localised particle motion and thereby remove particles from user-defined regions leading to higher particle contrast relative to the background. Hence, this impulse approach removes the need to solve the pressure^21,27,37^ or force^19,20^ optimisation problems which do not have obvious best solutions.

## Results

### Simulated impulse response

To understand the behaviour of particles under impulse we first conduct simulations using a dynamic time-domain model of the impact of multiple acoustic fields in a sequence and consider their combined effect. For details of the simulation see “Methods”.

The impulse exerted on a particle is defined as the force on a particle integrated over a given time. If each acoustic state *q* leads to a force $\mathbf {F}_{\mathbf {ac,q}}({\mathbf {x}}(t))$ on a particle at time dependent location ${\mathbf {x}}(t)$ the impulse $\mathbf {I_q}$ over a given small time step $\Delta t_q$ is given by Eq. (1).

1 $$\begin{aligned} \begin{aligned} \mathbf {I}_{\mathbf {q}}=\int _{0}^{\Delta t_q} \mathbf {F}_{\mathbf {ac,q}}({\mathbf {x}}(t)) dt \end{aligned} \end{aligned}$$

**Figure 1:**
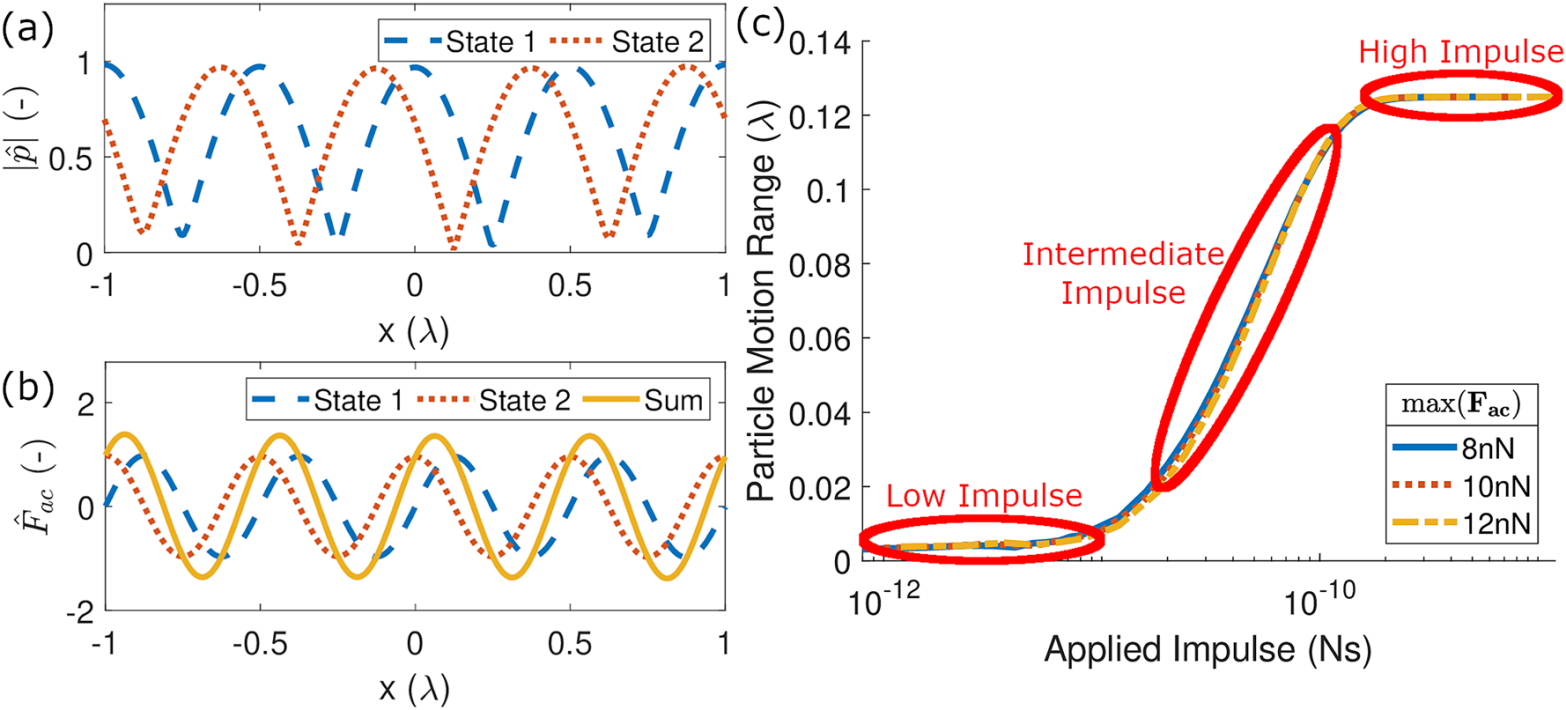
(**a**) The two normalised absolute 1D pressure fields, $|{\hat{p}}|$, with one source’s phase output offset from the other by $\frac{\pi }{2}$. (**b**) The normalised acoustic forces, ${\hat{F}}_{ac}$, generated by the pressure fields in (**a**) and the sum of these two fields. (**c**) The variation in particle motion per cycle vs the applied impulse, $I_{app}$, varied by both the maximum force and the switching time. The different lines indicate different maximum $\mathbf {F}_{\mathbf {ac}}$ values which the force field was normalised to and each force was applied with a $\Delta t_s$ range from 0.1 ms up to 100 ms. Note that the lines overlap, indicating that applied impulse produces a consistent effect regardless of which component is altered.

To understand how a particle moves as the impulse is changed, consider the scenario shown in Fig. 1a in which the pressure field is a simple 1D standing wave field with two states. Each state will be applied for an equal time period before switching to the other state. This is referred to as the switching time, $\Delta t_s$. There is a phase shift of $\frac{\pi }{2}$ between each of these two states resulting in a spatial shifting of the pressure nodes/antinodes by $x=\frac{\lambda }{4}$. A small particle in either of these pressure fields in isolation will experience an acoustic radiation force field obtained from Eq. 10 and shown in Fig. 1b. The range of the particle’s motion over $100\Delta t_s$ is plotted on the y-axis of Fig. 1c. The x-axis plots variation the applied impulse, i.e. $I_{app}=\text {max}(\mathbf {F}_{\mathbf {ac}})\Delta t_s$. This is chosen as the maximum acoustic force of each state and the time it is applied for are the two control variables that can be directly set by the user. Applied impulse is varied by changing both the switching time (along the x-axis) and the maximum force (different lines). As can be seen, the lines collapse onto a single line demonstrating that applied impulse is a variable which produces a consistent effect regardless of which component is altered.

In the high impulse regime shown in Fig. 1c the particle oscillates between the two stable equilibrium positions that occur at each of the two states as it has sufficient time to move to that equilibrium state for the given force field. This represents slow state switching or high forces. This high impulse regime has been used by various authors to dynamically manipulate particles along specified paths^13,38,39^.

In the low impulse regime shown in Fig. 1c the particle moves to and stays at the single equilibrium that emerges as the sum of the two force fields. This is because the switching time is small relative to the particle response time for the force fields used. This means that the particle does not have time to follow the force vectors to their respective equilibria, as in the high impulse regime, but instead experiences an average over the response time. In the case shown in Fig. 1 at the equilibrium of the summed force fields the particle is exposed to a cycle of two forces of the same magnitude for the same time in opposite directions, meaning that the net force experienced is zero. When the particle is located away from the equilibrium one of these force fields will have a larger magnitude than the other meaning that it experiences a net force field towards the equilibrium. In Fig. 1c the particle begins a short distance from the equilibrium meaning it moves a short distance to the location and then remains there. Note that the switching time must remain significantly greater than the period of the acoustic excitation. For megahertz ultrasonic waves this is in the order of microseconds and is therefore orders of magnitude shorter than the particle response time.

The location of the equilibrium in the low impulse regime depends on the relative magnitude of the forces employed and the time for which they are presented. The general effective low impulse force field, $\mathbf {F}_{\mathbf {sum}}$, of a sequence of *Q* applied fields is therefore the sum of the component acoustic force fields $\mathbf {F}_{\mathbf {ac,q}}$ weighted by the fractional time they are present,

2 $$\begin{aligned} \begin{aligned} \mathbf {F}_{\mathbf {sum}}=\frac{\sum _{q=1}^{q=Q} \mathbf {F}_{\mathbf {ac,q}} \Delta t_{q}}{\sum _{q=1}^{q=Q} \Delta t_q} \end{aligned} \end{aligned}$$

where the numerator is the sum of the impulses exerted and the denominator is the total time taken to cycle through the *Q* states. If, as in this paper, a constant $\Delta t_q$ is used this can be simplified to,

3 $$\begin{aligned} \begin{aligned} \mathbf {F}_{\mathbf {sum}}=\frac{\sum _{q=1}^{q=Q} \mathbf {F}_{\mathbf {ac,q}}}{Q}. \end{aligned} \end{aligned}$$

Between these two extreme impulse regimes is an intermediate regime which is characterised by some particle oscillation without the particle ever reaching the equilibrium of either state. In this regime the resulting particle motion is harder to predict as it is more strongly influenced by friction.

### Experimental realisation of low impulse patterning

**Figure 2:**
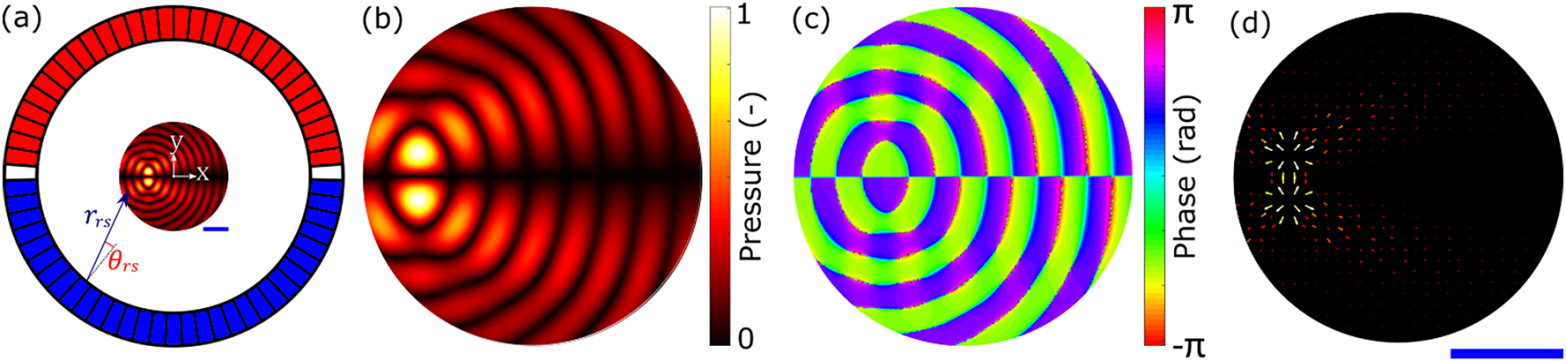
(**a**) Schematic of the circular phased array used in both the experiments and simulations. There are 64 phase and amplitude controlled elements around a circular chamber of 10.98 mm in diameter. The element colours indicate the phase offset for a twin trap, red and blue are $\pi$ apart and the white elements are turned off. There will also be focusing phases to account for the location of the trap relative to the centre defined by Eq. (6). (**b**) An enlarged view of the pressure field amplitude in the central 4 mm diameter region showing a twin trap offset from the centre in the x-axis. (**c**) The pressure field phase of the twin trap (on the same scale as (**b**)). (**d**) The resultant force field from the pressure field (on the same scale as (**b**)). The blue scale bar is 1 mm.

We now explore the potential of the low impulse regime for patterning and compare it to the alternative previous approaches of using a continuous wave field. An experimental 64-element circular array device shown in Fig. 2a was used to demonstrate the impulse patterning concepts. The area of interest in the centre was simulated, assuming continuous-wave excitation, and the enlarged field is shown in terms of the normalised absolute pressure in Fig.  2b and in terms of the phase in Fig. 2c. Figure 2d shows the simulated resulting forces on $90\mu m$ diameter spherical polystyrene microparticle.

In the first example of low impulse patterning, a twin trap is used as the building block for the creation of a line feature. This is a type of acoustic trap consisting of two high pressure regions either side of a low pressure centre^26^. It is created by focusing the array at the desired trapping point and then inverting the phase of transducers on one side of the trapping axis to create a low pressure trapping region in the centre where the two phases cancel out. The low pressure cancellation region can be seen in Fig. 2b and the opposing phases in Fig. 2c; the sources inverted relative to each other are illustrated by the red and blue transducers in Fig. 2a. See “Methods” for more details.

**Figure 3:**
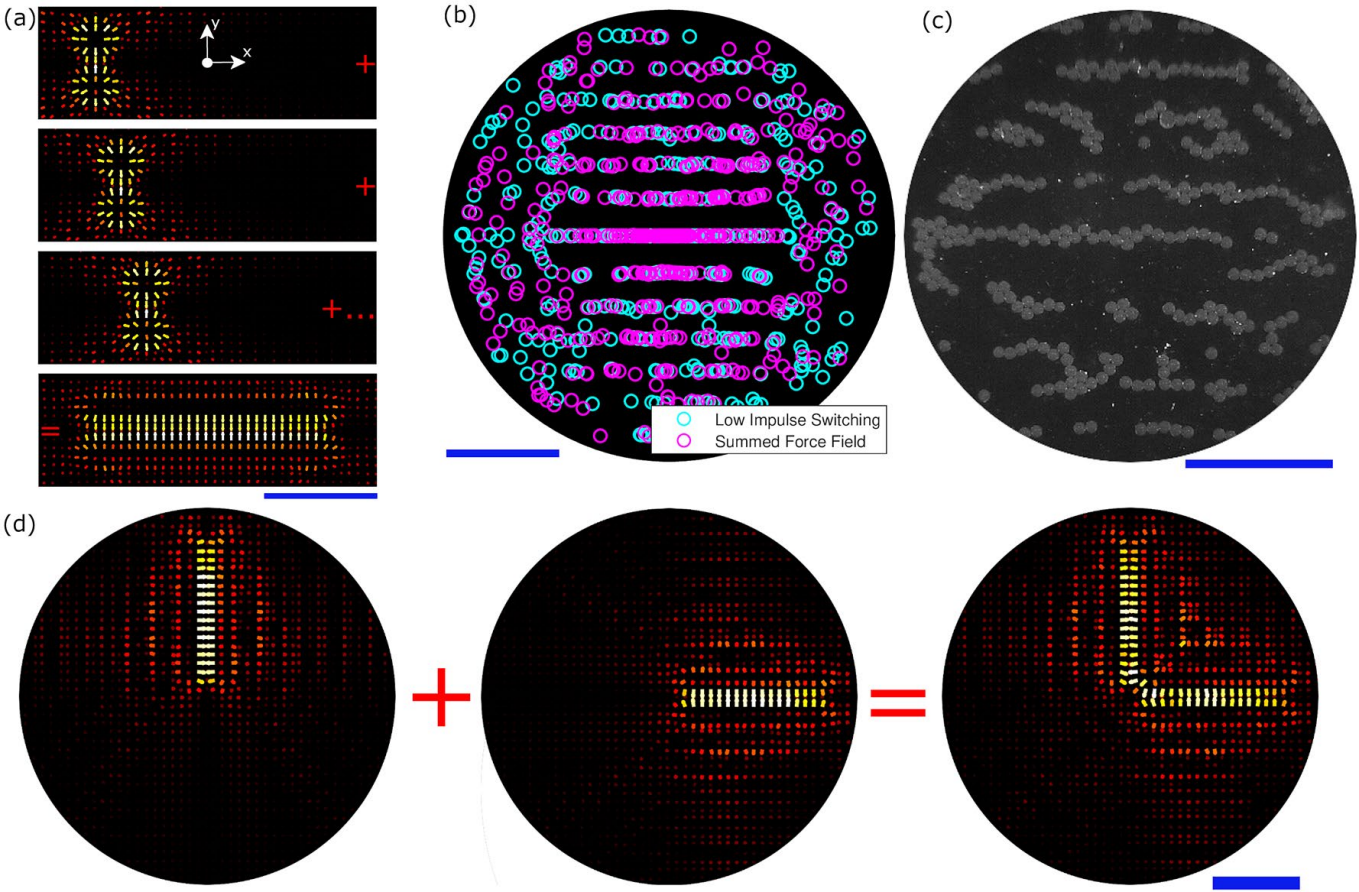
(**a**) Force field summation in the low impulse regime. Each arrow scales both length and colour to the magnitude of the vector at the origin point. A 2 mm line is formed from 10 twin traps. The xy axis is the same as in Fig. 2a with the origin representing the centre of the array. (**b**) demonstrates a simulated comparison of the particle end locations after being exposed to the switched force field or the summed vector field shown in the bottom panel of (**a**). Both fields had a maximum force of 10 nN and in the switched case the $\Delta t_q=1$  ms. (**c**) shows the application of a low impulse switched field to real particles. (**d**) demonstrates the simulated combination of two of these lines to create an ‘L’ shape. Each side of the L-shape is 1.5 mm long and made up of 8 twin traps (16 total). The blue scale bars are 1 mm.

Figure 3a shows the process of creating a line feature using a rapid sequence of twin traps. Each trap creates one of the component force states that, when combined, form a linear force field. The first twin trap is specified at $(-1,0)$ mm and the following twin traps then spaced at approximately $x=\frac{\lambda }{3}$ intervals to achieve the desired line length (an algorithmic spacing technique detailed in “Methods” was used to enable any line length to be specified, rather than being limited to $\frac{\lambda }{3}$ increments). In this case 10 twin traps are used to produce a 2 mm long line with the resulting force field shown in the bottom panel of Fig. 3a. Focused points and vortex traps were also explored as alternative building blocks for forming a line. The focused points were found to have a the same effect but required twice as many switching states. Vortex traps had a much smaller point-like trapping region and so also required more states to achieve a similar line effect.

Figure 3b shows the results of a time domain simulation illustrating the validity of force field summation in the low impulse regime. Two separate simulations of the force field in Fig. 3a were conducted on an initially random distribution of particles and the final locations are plotted. The cyan particles represent those which experienced the switched force field. The magenta particles experienced the constant weighted sum force field defined by Eq. 3 and shown in the bottom panel of Fig. 3a. Both simulations were run for 500 ms with the maximum force set to 10 nN. For the switched simulation, $\Delta t_q = 1$ ms, meaning the maximum available impulse by each field was $1 \times 10^{-11}$ Ns (i.e. within the low impulse regime). Good agreement between the switched field and constant summed field can be seen in terms of the resulting patterns. This is then further validated by Fig. 3c which shows a microscope image of this pattern created via low impulse switching with good agreement between the patterns.

Figure 3d shows how fields such as those seen in Fig. 3a may be added together as building blocks to create increasingly complex force fields. Here an ’L’ shape with 1.5 mm sides was formed from 16 twin traps (8 per line). As the number of component force fields increases, so $\Delta t_q$ must decrease such that maximum available impulse remains low. Figure 3a suggests that using a twin trap as the building block means that the forces due to each component field are localised to within a few wavelengths. However, this low impulse patterning process can be seen to create unwanted areas of force away from the main pattern, as indicated by the parallel line features away from the desired central line. Even in these simple shapes, the fields created by this approach are superior in form and more robust to those generated by pressure field optimisation for the same arrangement (see the supplementary information).

### Particle clearing and agglomeration

The experimental generation of a line feature shown in Fig. 3c gives good agreement with the simulated results but the image raises an obvious issue with recreating the fidelity of the desired pattern: the contrast of the desired particle pattern against the background. Here, we resolve this problem by using additional field states to clear away the particles from the undesired regions and create a single isolated line.

**Figure 4:**
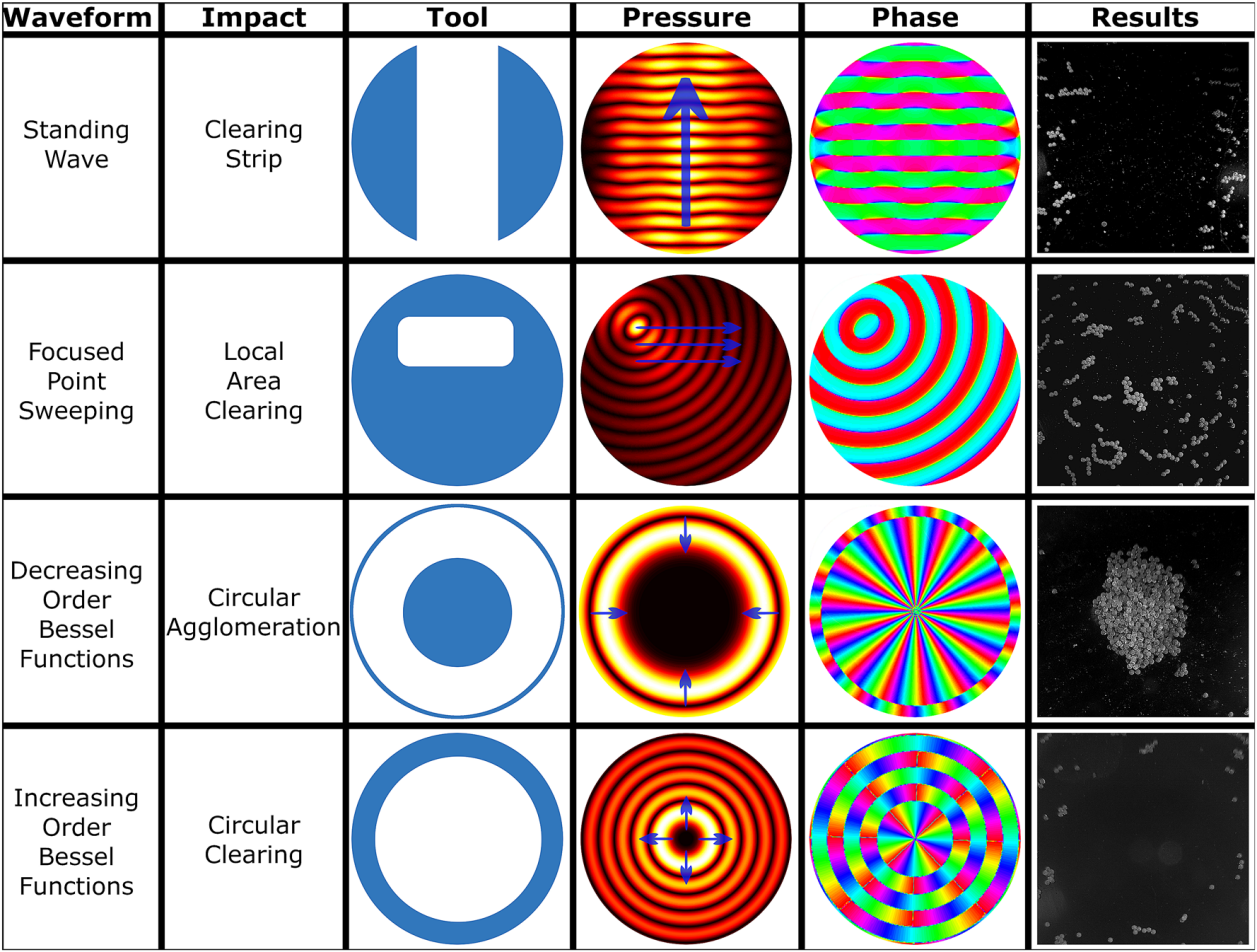
Diagrams of a selection of tools which can clear or agglomerate the particles. The results are microscope images of the impact after the technique is applied to a random distribution of particles. The standing wave conveyor belt allows a strip of particles to be cleared. Sweeping a focused point allows a specified area to be cleared. Cycling down the Bessel function order agglomerates the particles into a single clump. Cycling up the Bessel function order clears a circular area of the particles to the edge. Videos of the tools working can be seen in Supplementary Video 1.

The approach described here makes use of the intermediate and high impulse regimes to define a selection of clearing and agglomerating *tools* that are shown in Fig. 4. Note that the approach is not a single solution to clear and agglomerate the particles, rather a number of tools that can be deployed in differing ways depending on the need. Indeed, it emerges that these tools can be deployed in a variety of sequences to achieve the same end result. Each will be briefly explained here with further details in “Methods”.

The standing wave acts as a particle conveyor belt, transferring particles in a straight line from all locations along its path to one end. This clears a strip across the device. It uses a looped progression of standing waves with different phases in the high impulse regime to sweep the particles to the edge of the device.

The focus point sweeping is used to clear a small local area. By moving a focal point (i.e. a 0th order Bessel function shaped field) along a line, particles are pushed away from the focus. By performing this operation in the intermediate impulse regime the path followed by the focus and a small area around it can be cleared and doing this along a number of parallel lines allows a larger area to be cleared.

Changing the angular order of the generated Bessel function shaped field changes the size of the low intensity central region^35,40^. By operating in the high impulse regime and progressively reducing this order the particles are forced inwards until they form an agglomeration in the centre of the resulting field. By doing the opposite (progressively increasing the order) the central area can be cleared of particles.

**Figure 5:**
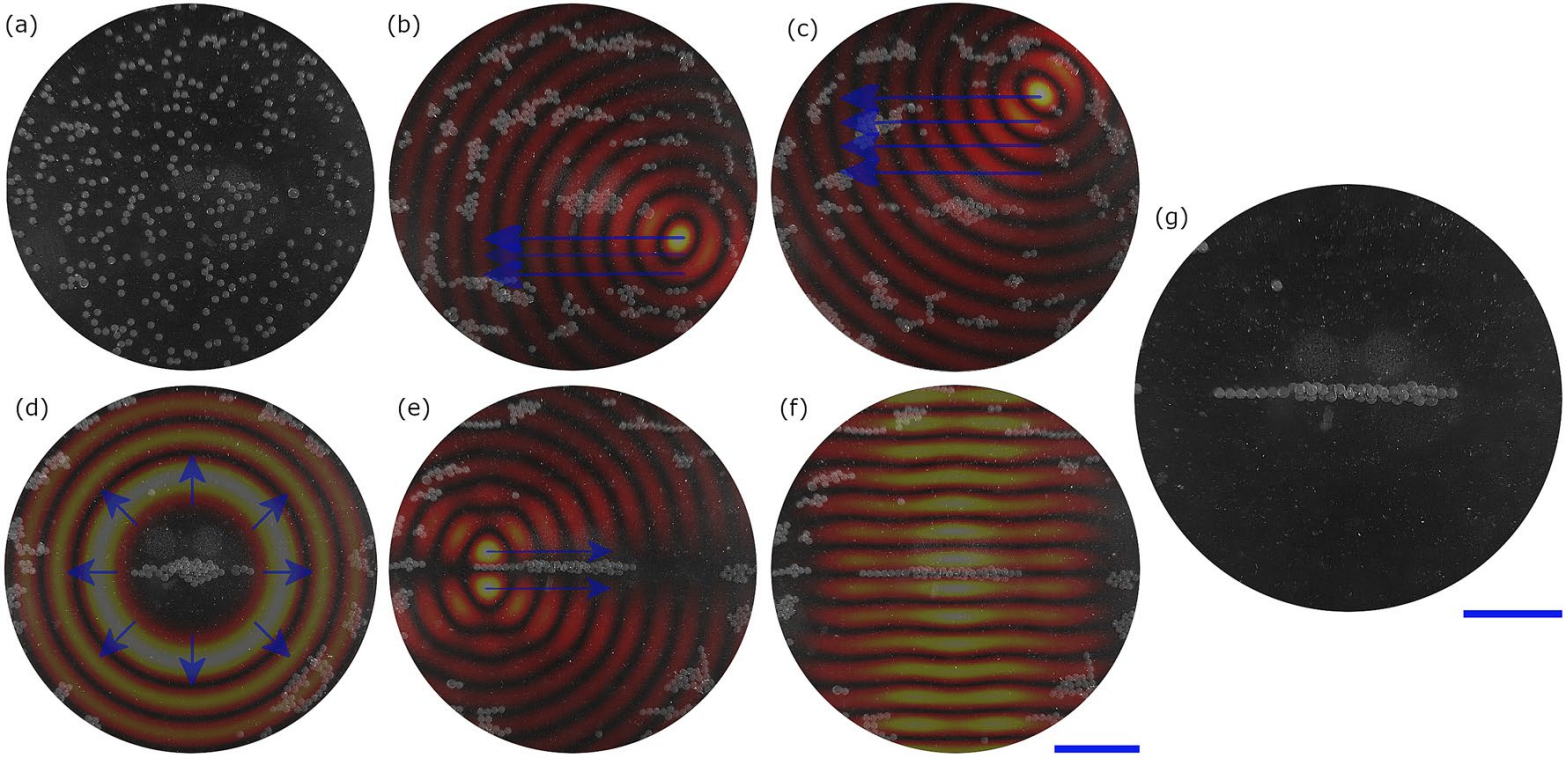
The process for forming a line combining clearing techniques and multiplexing. Starting with a random particle distribution in (**a**). A focused point sweep is then used “below” the line, with the results shown in (**b**). This is repeated “above” the line, clearing the area and agglomerating some particles into the line, resulting in (**c**). Increasing order Bessel functions are then cycled up resulting in (**d**). Finally the line is shaped and reinforced through a combination of switching low impulse twin traps (**e**) and applying a static standing wave (**f**), resulting in the line with a cleared area around it, as shown in (**g**). The blue scale bars are 1 mm. This process is shown in action in Supplementary Video 2.

The patterning steps shown in Fig. 3 can now be combined with the clearing tools shown in Fig. 4 to create localised patterns of particles with a high contrast to the background. Figure 5 shows the process for forming a single line in a cleared workspace. It should be stressed that the sequence of operations described in detail below is one of a number of possible routes to the same result. The first stage is local area clearing below (Fig. 5b) and above (Fig. 5c) the line. Increasing order Bessel functions are then used to clear the surrounding area (Fig. 5d). Between each step and at the end the line is reinforced using twin traps (Fig. 5e). At the end the line is further reinforced using a static standing wave (Fig. 5f). The final isolated line is shown in Fig. 5g. Further details are given in the “Methods” section.

**Figure 6:**
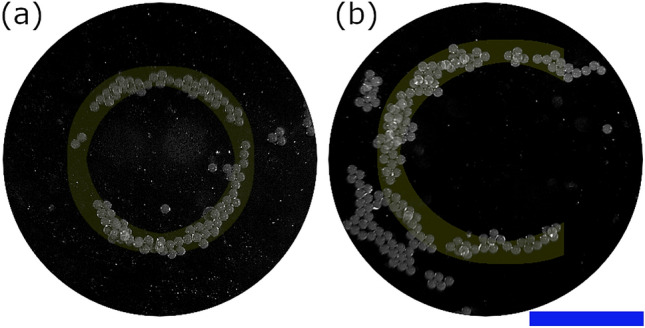
Example shapes formed by combining patterning in the low impulse regime with higher impulse clearing operations. (**a**) shows a circle formed by switching between states consisting of a 5th and 9th order Bessel-shaped field. (**b**) shows an C-shape formed by a collection of twin traps The blue scale bar is 1 mm. For forming of (**a**) see Supplementary Video 3.

Figure 6 shows further experimental examples of patterns that have been achieved with the impulse technique. In both cases the starting point is a random distribution of particles similar to Fig. 5a. To achieved the circle shown in Fig. 6a a set of high impulse increasing and decreasing order Bessel-shaped fields with a common origin point are first used to clear and agglomerate the particles and form an approximate circle. The circle shown is then formed in the low impulse regime by switching between 5th and 9th order Bessel-shaped fields. To form the ’C’ shape in Fig. 6b a set of high impulse increasing and decreasing order Bessel-shaped field with a common origin point are used to first create an approximate circle and then a set of increasing order Bessel-shaped fields with an offset origin clear the gap on the right hand side. The ’C’ shape is then formed in the low impulse regime by a collection of twin traps.

### Further pattern complexity and dynamic manipulation

The patterning processing described in the previous sections is order dependent and so at present different desired patterns each require a bespoke clearing regime. Very different shapes (e.g. the circle and the line) require different sequences of clearing steps to be formed directly. However, through extensive experimentation we have observed that typically there are multiple routes to the same end result. One aspect that emerges is that complex shapes can be formed from simple building blocks. This means that rather than creating a new sequence for a different shape or location, an existing patterning and clearing process may be employed to first produce a simple shape, such as a line, and this is then transformed into the desired shape. This is achieved by slowly moving between sets of low impulse force fields such that the net force field moves slowly enough to keep the particles in the desired place.

**Figure 7:**
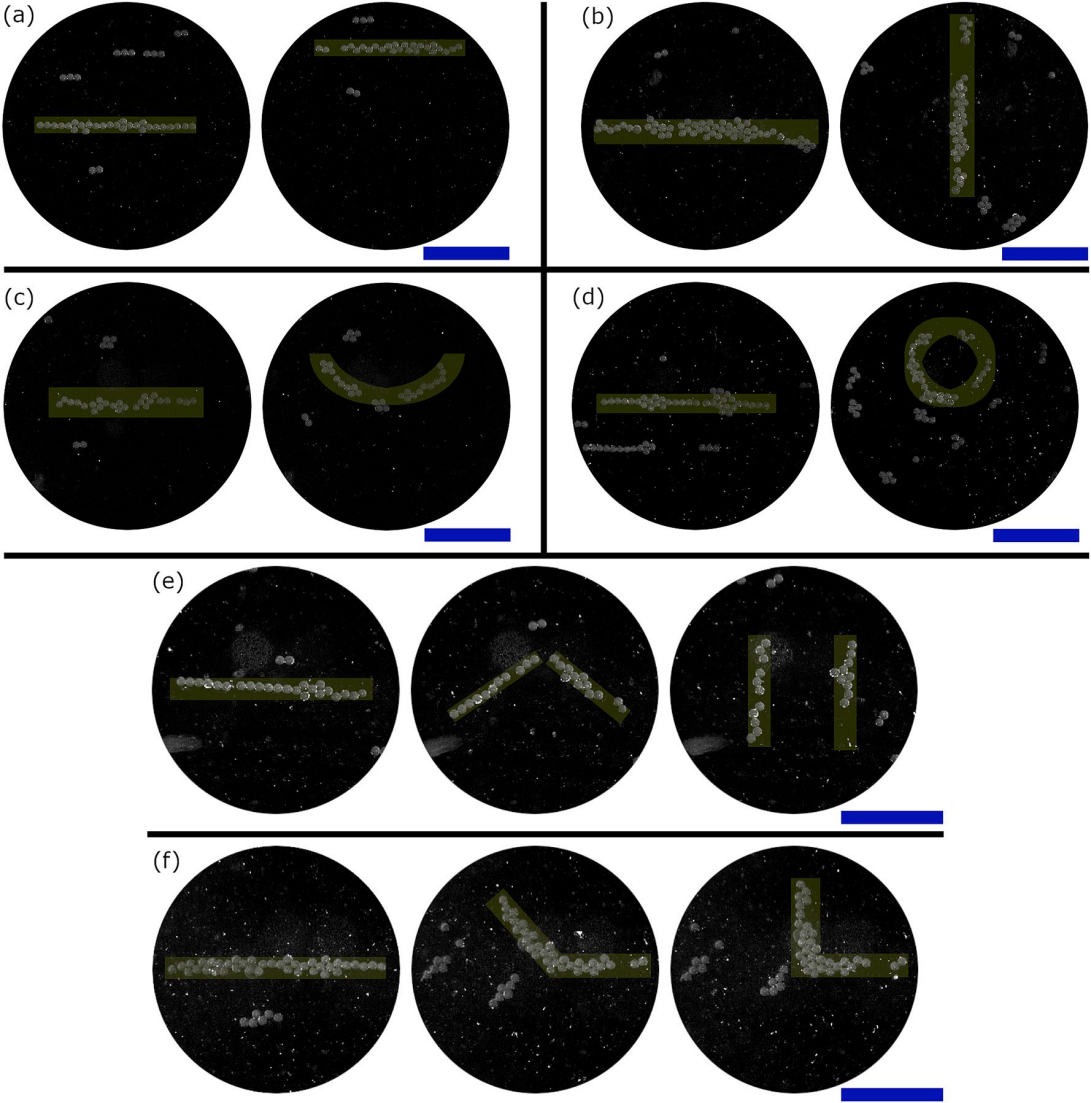
By incremental movement of the switched low impulse force fields the line can be dynamically (**a**) moved, (**b**) rotated or reshaped into (**c**) an arc or (**d**) a circle. A line can also be manipulated in two halves. (**e**) shows the line being split into two half lines and rotating in opposite directions while (**f**) shows one half line being rotated whilst the other half is held in location to form an ‘L’ shape like that shown in Fig. 3d. The blue scale bar is 1 mm. These operations can be seen happening in Supplementary Video 4.

Figure 7 illustrates a number of reshaping and translation operations that can be applied to a pre-created line. (a) shows 1 mm of linear motion perpendicular to the line, (b) shows $90^o$ of clockwise rotation, (c) shows deformation of the line into an arc of 3 mm radius by movement of the twin traps into an arc and (d) shows deformation into a circle of approximately 1 mm radius where the twin traps reshape the line into and arc and then in a final step change to a low impulse regime switching of a 2nd and 6th order Bessel-shaped field. Once formed, the line feature can also be subdivided for further manipulation, (d) demonstrates the process of dividing the line in two and rotating it in opposite directions while (e) demonstrates keeping the halves connected, rotating one half and holding the other in place to form an ‘L' shape. Further details are given in “Methods”.

This is an illustrative set of basic examples of the potential of the impulse control technique. These examples start from the formation of some relatively simple patterns, such as a line to a circle, which then act as building blocks from which more complexity can be generated as needed. This reshaping approach has the benefit that the clearing operation can be optimised for the simple shape and is then retained for the more complex shape. Hence, it provides a reliable route to high fidelity patterns that is suitable for future automation.

**Figure 8:**
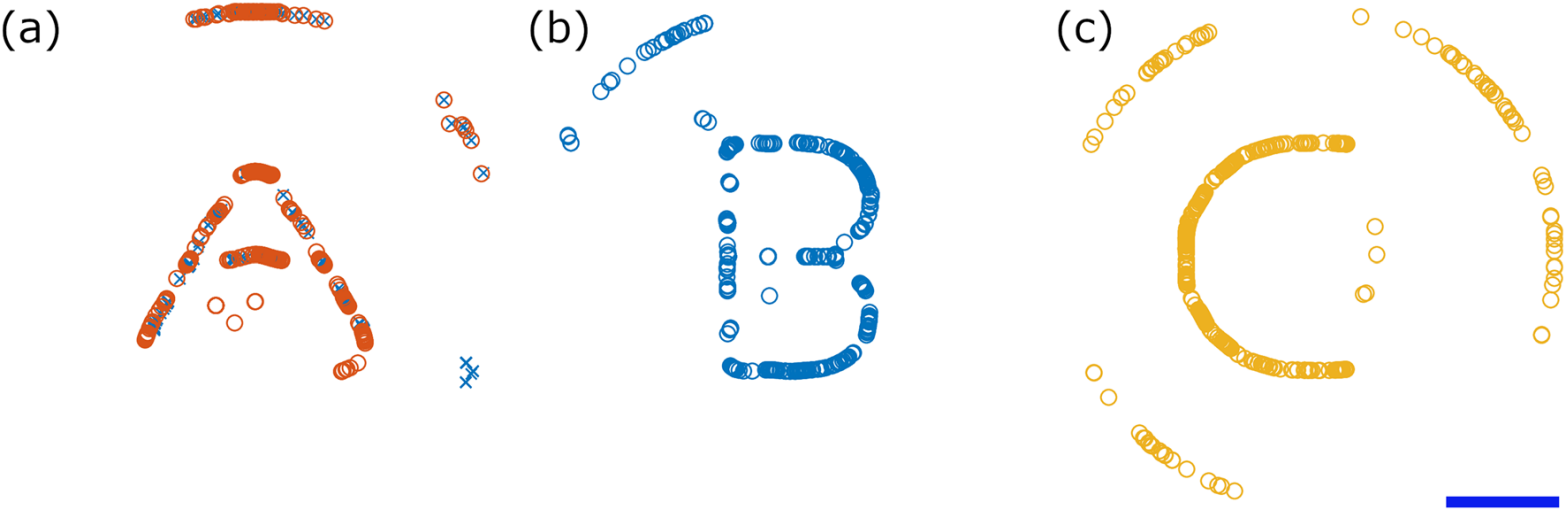
Simulated examples of complex particle patterns possible with the presented patterning and clearing techniques. A systematic clearing approach has been applied to the circular particle locations in all figures. (**a**) has also had custom clearing applied to remove any remaining particles with the final location indicated by the blue crosses. This was primarily achieved using an intermediate impulse vortex trap to move each particle away from the shape, then a standing wave to pull them further away. Note that the particles have been moved away but there is minimal disturbance to the coherence of the pattern. The blue scale bar is 1 mm. See Supplementary Video 5 for animations of these processes.

The experimental demonstrations of Figs. 5, 6 and 7 should be viewed as examples of the possibilities of impulse pattering methodology. As discussed, the patterns described are building blocks and are therefore expandable. Figure 8 shows simulated patterns of particles cleared into complex letter shapes using our time domain simulation on an 64-element array device with the same geometry as used in this paper (see Fig. 2a) but with instantaneous switching and perfect phase and amplitude control, as opposed to our experimental device which has time limited switching and less accurate phase and amplitude control (see “Methods” for more details). The experimental work presented in this paper is therefore provided as a proof of the concepts proposed but these simulations give a better picture of the technique’s ultimate potential.

Initially, the basic letter patterns were formed in the low impulse regime by use of twin traps that trace out the required shape. This created the pattern but resulted in poor pattern contrast, e.g. for the A-shape $60.1\%$ of the particles are not in the desired shape or at the edge of the manipulation region. By instead starting with clearing techniques the particles in unwanted locations can be removed, resulting in a reduction to only $0.5\%$ of particles outside the desired pattern for the A-shape. A summary of the data for all shapes is given in the supplementary information as well as videos of the pattern formation. Figure 8a also shows individual particles that are removed as a final stage (the blue crosses). Here a carefully impulse controlled vortex trap at the high end of the intermediate regime was able to move individual particles without disrupting the main pattern. A localised standing wave conveyor belt then moves them to the edge. This demonstrates that precision manipulation of individual particles is possible in this ideal device.

## Discussion

We have shown that the motion of particles under changing acoustic force fields is dependent on the impulse applied by the fields. We have then demonstrated that arbitrary force fields can be formed by combining multiple fields which act as building blocks. The impulse behaviour can be broken down into three key regimes. In the low impulse regime the force field can be considered equal to the time weighted sum of the force fields from each constituent pressure field which can be used for complex particle patterning. With this regime it was shown possible to construct and manipulate simple shapes such as lines and circles. It was also shown possible to morph these simple shapes into more complex asymmetric shapes such as an L-shape. We have also identified a number of particle clearing tools. These enabled the contrast of the patterns to be improved by removing many of the particles that remain in undesired locations. In the high impulse regime particles move to the force equilibrium position of the field each time it changes, but this can be done progressively for larger ranges of motion which can be used to clear large areas of the particle field. Finally, in the intermediate region between these two the particle begins to move towards the steady state but does not quite reach it. This can be used for precise clearing of particles from local regions.

Using impulse control we were able to form patterns of particles that would not be possible with a single continuous wave field. By controlling the time each component field is applied, as well as the phase and amplitude, we can use the impulse to move beyond the apparent geometric limitations of our devices and into more complex and precise control and patterning of particles.

The experimental realisations shown in this paper were limited by the device performance, the maximum switching speed in particular. Future devices will make it possible to create more complex pressure fields as the basic building blocks and clearing tools. The array device could also be combined with a hologram encoded with a specific building block. The phased array device could then augment this initial pattern, for example by applying various clearing operations, thereby producing a high contrast final pattern.

## Methods

### Simulating a particle in time

To model impulse-based manipulation on particles we must simulate their behaviour in an acoustic field over time. We use a 2D Huygens’ principle model to predict the acoustic pressure field and the Gor’kov potential to calculate the acoustic radiation force on particles that are small relative to the acoustic wavelength. Note, for simplicity and consistency, all particles used have a positive acoustic contrast meaning that they move to the low-pressure nodes of the steady-state acoustic pressure field^41^.

As an example we start with a 2D twin trap created within a circular phased array device as shown in Fig. 2a. This device allows a wide range of 2D manipulation possibilities and is later explored experimentally. The pressure field, *p* is calculated via matrix propagation^42^ given the known amplitude and phase of the source elements, $a_0$, as in Eq. (4). The transfer matrix, $H_{rs}$, is calculated by Eq. (5) where $S_w$ is a scaling factor relating a fibre optic hydrophone (Precision Acoustics, UK) pressure measurement within the array to the simulation, *a* is the element width, $k=2\pi /\lambda$ is the wavenumber, and $\lambda$ is the wavelength. The distance $r_{rs}$ and angle from the element normal $\theta _{rs}$ are illustrated in Fig. 2a.

4 $$\begin{aligned} \begin{aligned} p=H_{rs}a_0 \end{aligned} \end{aligned}$$

5 $$\begin{aligned} \begin{aligned} H_{rs}= \frac{S_w e^{-kr_{rs}}}{2\pi \sqrt{r_{rs}}} \text {sinc} \left[ \frac{a\pi ^2 \text {sin}(\theta _{rs})}{\lambda } \right] \end{aligned} \end{aligned}$$

Equation (6) shows how the phase, $\phi$, on the $n{\text {th}}$ element in the circular array is calculated^17^. Here, *m* is the angular order (or helicity) of the Bessel function, *N* is the total number of elements in the array and $r_n$ is the distance from the element to the focal point.

6 $$\begin{aligned} \begin{aligned} \phi _n = \frac{2m\pi (n-1)}{N}-kr_n \end{aligned} \end{aligned}$$

A twin trap is the sum of two fields: a focusing field (which determines the location) and a cancellation field (which determines the orientation). To generate a twin trap with a desired location and orientation the transducer phases for each of these fields are calculated separately and then summed. The focusing field phases are determined by calculating a 0th order Bessel function at the desired location by setting $m=0$ in Eq. (6). To calculate the cancellation field an axis of desired cancellation (intersecting with the focus location) is selected and the phases on either side of it are offset by $\pi$ radians. Figure 2a shows this set of phases where the cancellation axis is the x-axis. Here the red elements are offset by $\pi$ from the blue elements. The white elements represent those directly on the cancellation axis, which are set to have zero amplitude.

We now need to calculate the particle behaviour under a force field. The location of a particle at time *t* is defined by a position vector $\mathbf {x}_{\mathbf {t}}$. The motion is calculated incrementally with a sufficiently small time step $\Delta t$ such that the force, $\mathbf {F}_{\mathbf {e}}$ and velocity ${\dot{\mathbf {x}}_t}$ can be considered constant over the time step. Integrating the acceleration gives the location after each time step as Eq. (7).

7 $$\begin{aligned} \begin{aligned} \mathbf {x}_{\mathbf {t}+\varvec{\Delta } \mathbf {t}}=\int _{t}^{t+\Delta t} \int _{t}^{t+\Delta t} \frac{\mathbf {F}_{\mathbf {e}}({\mathbf {x}},{\dot{\mathbf {x}}})}{m_v} dt dt = \mathbf {x}_{\mathbf {t}} + {\dot{\mathbf {x}}}_{\mathbf {t}} \Delta t + \frac{\mathbf {F}_{\mathbf {e}}({\mathbf {x}},{\dot{\mathbf {x}}})\Delta t^2}{m_v} \end{aligned} \end{aligned}$$

To account for entrained flow we use a virtual mass, $m_v$ calculated by Eq. (8) where $r_p$ is the particle radius, $\rho _p$ is the particle density and $\rho _0$ is the fluid density^43^.

8 $$\begin{aligned} \begin{aligned} m_v = \frac{4}{3}\pi r_p^3\left( \rho _p + \frac{\rho _0}{2}\right) \end{aligned} \end{aligned}$$

The inertial force is then balanced by the acoustic force, $\mathbf {F}_{\mathbf {ac}}$, and the drag force, $\mathbf {F}_{\mathbf {d}}$, as seen in Eq. (9). Note that the neglected forces such as friction are discussed later in this section.

9 $$\begin{aligned} \begin{aligned} \mathbf {F}_{\mathbf {e}} = \mathbf {F}_{\mathbf {ac}}({\mathbf {x}})-\mathbf {F}_{\mathbf {d}}({\dot{\mathbf {x}}}) \end{aligned} \end{aligned}$$

The acoustic force field on small particles in a given pressure field is calculated by the gradient of the Gor’kov potential^41,44^ as is shown in Eq. (10). $U^{rad}$ is the Gor’kov potential as defined in Eq. (11) where, $\langle p^2 \rangle$ and $\langle v^2 \rangle$ are the mean squared acoustic pressure and velocity, respectively, $\kappa =1/(\rho c^2)$ is the compressibility and the subscripts 0 and *p* refer to the host fluid and particle respectively. The material-dependent pre-factors are given by $f_1 = 1 - \kappa _p/ \kappa _0$ and $f_2 = 2(\rho _p - \rho _0)/(2\rho _p+\rho _0)$. Throughout this paper we assume the host fluid is water and the particles are $90\mu m$ diameter polystyrene spheres, hence $c_0=1500\,\text{m}/\text{s}$, $\rho _0=997\,\text{kg}/\text{m}^3$ (at 25$^o$C^45^), $\mu _0 = 0.89\, \text{mPa} \, \text{s}$, $c_p=2047\,\text{m}/\text{s}$ and $\rho _p=1050\,\text{kg}/\text{m}^3.$

10 $$\begin{aligned}{}&\begin{aligned} \mathbf {F}_{\mathbf {ac}}=-\nabla U^{rad} \end{aligned} \end{aligned}$$

11 $$\begin{aligned} U^{rad} = \frac{4 r_p^3}{3} \left[ f_1 \frac{1}{2} \; \kappa _0 \; \langle p^2 \rangle - f_2 \frac{3}{4} \; \rho _0 \; \langle v^2 \rangle \right] . \end{aligned}$$

The drag force is calculated from Eq. (12) with the coefficient of drag given by Eq. (13)^46^.

12 $$\begin{aligned} \mathbf {F}_{\mathbf {d}}=\frac{\pi r_p^2 \rho _0 {\dot{\mathbf {x}}}_{t} C_{d}}{2} \end{aligned}$$

13 $$\begin{aligned} C_d = \left( \frac{24}{Re} \right) \left( 1+\frac{3}{16}|Re|\right) ^{0.5} \end{aligned}$$

In the above, we have assumed that the acoustic and drag forces dominate for our system and it is later shown that this gives good agreement with our experimental results. Some other forces have been neglected as they do not have a significant impact in this context. Acoustic streaming forces become dominant for particles below a critical size to wavelength ratio^47^, but the relatively large size of the particles used in this paper make this effect small. As our particles rest on the bottom of the chamber there will be friction forces present. However, these are relatively low because the particle density is close to water (i.e. 5$\%$ density contrast) meaning that buoyancy acts against gravity. This reduces the normal force between the particles and the surface and hence the friction. Finally, close-range inter-particle forces mean that once the particles have agglomerated they have a tendency to remain in contact to minimise their surface tension against the water. As we start the various patterning operations from an initial random distribution (where there is little or no inter-particle force) and then form the patterns from which the particles are not removed this does not affect the accuracy of our model. It simply means that once our particles have moved into the desired pattern they tend to stay there, i.e. the patterning process is one-way. If we were using pre-agglomerated clusters these inter-particle forces would become an important factor to add to the model.

### Experimental arrangement

The experiments were conducted in a 64-element circular phased array with a diameter of 10.98 mm, as illustrated in Fig. 2a. The elements are designed to emit the ultrasound into a central chamber where the patterning takes place^17^. The transducers are coated in a matching layer and have an absorbing backing so that they are non-reflective. This device operated at 2.35 MHz and, as the central chamber is filled with water, the operating wavelength $\lambda = 0.644 \, \text{mm}$. Sinusoidal electrical signals were sent to the array elements from a custom built 64 channel driver system and each channel had independent phase and amplitude control. The fastest update time for the custom built driver unit was 0.074 seconds with a standard deviation of 0.003 seconds and this limited the maximum field switching that could be achieved.

A microscope cover slide (thickness = 0.13 mm) was sealed to the bottom of the device with silicone sealant to contain the water and allow observation. The cover slide was coated with a WD-40 PTFE Dry Lubricant Spray (WD-40 Company, USA) to decrease friction. The particles used for patterning were $90\mu m$ diameter Fluoresbrite polystyrene particles (Polysciences Inc., USA). The particle solution in the central chamber was diluted to reduce particle density to the desired level with deionised water and a drop of a household surfactant was added to reduce inter-particle forces.

The device was calibrated using a fibre optic hydrophone (FOH) (Precision Acoustics Ltd, UK). A point scan of each element output was taken at the centre of the chamber to measure phase and amplitude and a calibration was applied to the excitation signal to correct for any observed variation and hence achieve the desired phase and amplitude outputs.

### Placement of twin traps in a line

It was found that the maximum spacing spacing for optimal line creation was $\le \frac{\lambda }{3}$. However, to allow arbitrary spacing, a short algorithm was written which would take line length as the input and calculate the number of twin traps, $N_{TT}$, by Eq. (14). The traps would then be equally distributed along this line.

14 $$\begin{aligned} N_{TT}= round \; up \left( \frac{Line \; length \times 3}{\lambda } \right) + 1 \end{aligned}$$

### Particle clearing and agglomeration

The tools shown in Fig. 4 are explained in detail here. Note that the conditions given are the result of iterative experimental testing with the device used. Similar iteration will be required for different devices.

The standing wave, acting as a particle conveyor belt, clears a strip across the device. It uses a looped progression of three standing waves, each separated by a phase offset of $\frac{2\pi }{3}$ to move the particles a short distance, eventually sweeping the particles to the edge of the device. Each field is applied in the high impulse regime with the maximum experimental input voltage (to maximise force) of 20 Volts and a time period of 2 seconds [20 V, 2 s]. This meant that the particles settled into that state before the next is applied, moving them progressively in a single direction. Outside of the standing wave field the force magnitude is sufficiently small to not move the particles. The gradient of the pressure from the centre of the conveyor belt to its edge pushes some particles outwards, further adding to the clearing effect. In the experimental device, the standing wave field is generated by activating sub-apertures of opposing elements. In the example the sub-apertures included 11 elements on each side (22 total). In addition to the phase offset of $\frac{2\pi }{3}$ between each step (added on one side and subtracted on the other) a phase shift of $kr_{sw}$ is applied to each element where *k* is the wavenumber and $r_{sw}$ is the distance from the element to the wavefront of the conveyor belt, thereby correcting for the curvature of the array and creating a plane wave at the desired location.

Focused point sweeping is used to clear a small local area. By moving a focal point (i.e. a 0th order Bessel function shaped field) along a line, particles are pushed away from the focus. By performing this operation in the intermediate impulse regime the path followed by the focus and a small area around it can be cleared. By repeating this sequentially along a number of parallel lines in a progressive sequence a larger region is cleared. For the functionality to work well it was found to be important that the direction of the sweeping remained constant, otherwise particles were cleared by one step and drawn back into the cleared areas in the next step. Hence, this operation required the most fine-tuning of all the techniques. If the acoustic force (controlled via the input voltage) applied is too low then the static friction will prevent the particles from moving altogether meaning there is a lower bound on the useful force which can be applied. The impulse must also not be too high or else the lower amplitude force rings of the Bessel shape cause unwanted motion away from the focus. In the example shown a step size of 0.1 mm = 0.16$\lambda$ was used for the spacing both of the points along each cleared line and the centre of the sweeping lines from each other. To achieve the precision necessary the minimum possible delay, $\Delta t =0.074$ s for our experimental equipment, was applied and the voltage was varied experimentally until the desired clearing effect was observed. A typical arrangement was [7 V, 74 ms], but the best values depended on the experimental conditions.

Changing the angular order of the generated Bessel function shaped field changes the size of the low intensity central region^35,40^. By operating in the high impulse regime [20 V, 1 s] and progressively reducing this order (i.e. *m* in Eq. 6) the particles are forced inwards until they form an agglomeration in the centre of the resulting field. In simulation this was possible with a single repetition however in practice multiple repetitions were found to produce more compact agglomerates as the outer lobes of the Bessel function shape also act as conveyor belts inwards once the initial highest pressure region had passed. It was also beneficial to change the chirality (i.e. the handedness) of the vortex between repetitions to provide more uniform agglomeration from all directions. This meant that during one cycle of angular order decrease the phase increases in a clockwise direction, and in the next in the anti-clockwise direction. This change in chirality was thought to lessen the impact on the force field of differences in element output, making the effect more repeatable. It is also worth noting that reducing the order such that the central low amplitude zone was smaller than the size of the agglomeration sometimes caused the agglomeration to break apart. We believe this effect was caused by the forces alternating in direction beyond the first high intensity ring. Hence it is preferable to use Bessel orders that lead to a slightly larger than intended central low amplitude zone. Increasing the order of the Bessel function shape results in the reverse effect to decreasing the order, allowing a central region to be cleared. Again, this was found to produce the best clearing effect with repetitions in the high impulse regime. Both of these Bessel function techniques can be used off-centre by the addition of suitable focusing delays as shown in Eq. 6. However, it was found that away from the centre more repetitions were needed to obtain the same effect. This is thought to be due to weaker forces away from the centre in the experimental device.

### Clearing a single line

The first stage is local area clearing using focused point sweeping (Fig. 5b and c). Initially the area below the line is cleared with a unidirectional raster scan of a focus of intermediate impulse [9 V, 74 ms]. Then the area above the line is cleared with lower intermediate impulse (to avoid drawing particles into the region) which, due to the lower impulse, must be repeated twice to achieve full clearing [7 V, 74 ms]. This serves to agglomerate the particles into the rough line shape. Next a line of twin traps are used to initially form the line, as shown in Fig. 3a. At this stage the twin traps are also applied in the intermediate impulse regime [10 V, 74 ms] and used in an order which sweeps them in the opposite direction to the previously used focused point clearing. This is to counter the tendency of the local clearing to push the agglomerated particles in their swept direction. Now that the local area has been cleared in an approximately circular region around the line, increasing order Bessel function shaped fields can be used to enlarge the cleared area. Bessel functions from order 9 up to 20 were used for this purpose [12 V, 300 ms]. A sequence of alternating chirality of vortices were used for uniform clearance. At intervals throughout the clearing a intermediate impulse twin trap line [10 V, 74 ms] was applied to avoid the emerging line becoming too distorted. Once the particles have been cleared the line is once again reinforced with twin traps [10 V, 74 ms]. Finally a single high impulse standing wave field [20 V, 30 s] with the low pressure region aligned with the particle line is used as a supplement the twin trap line. Here there is no updating of the field to sweep the particles away, instead the static field reinforces the line and helps obtain a more even distribution of particles along its length, as shown in Fig. 3f. Note that this field is only useful for reinforcing the single line now that the particles in close proximity have been removed.

### Line deformation

The reshaping operations shown in Fig. 7 all used an impulse in the low impulse regime [8 V, 74 ms], cycling through multiple repetitions of the applied total force field to incrementally alter shape or position. The line was moved in small increments: 0.1 mm per step for motion (a), $5^o$ for rotation (b). The deformation of a line into an arc or circle was also achieved by small incremental changes in the radius of the arc whilst maintaining a 2 mm length. For each of these steps the radius of the arc was also equal to the distance to the centre of the arc from the original line, i.e. the centre of the line stayed in place while the edges were curved. As the changes in the amount of deformation became greater as the radius got shorter it was deformed by the following steps: between 5 mm and 3 mm 0.5 mm/step, between 3 mm to 1 mm 0.25 mm/step, at less than 1 mm 0.12 mm/step. The fields were applied with the twin traps sweeping in opposing directions in each step, for example in the line movement the first step up would sweep in the +x direction, then the next in the -x direction, then the next in the +x direction and so forth. It was observed that twin trap sweeping in a single direction resulted in a gradual pattern shift in the direction of the sweep. The chirality of the Bessel-shaped fields used to the generated circle in Fig. 7d was also alternated, as this was found to more evenly distribute the particles around the circle.

In the cases shown in Fig. 7e and f, the approach is operated in the low impulse regime [8 V, 74 ms] and switches between patterns of twin traps. As above, this process was performed incrementally in a series of steps in which the twin traps were moved towards their target in 5° increments.

### Simulated pattern forming

To generate the patterns in Fig. 8$F_{ac,max}=10$nN and $\Delta t_q = 1$ ms were used, yielding an applied impulse of 10pNs for each switching state. Here, the letters ’A’, ’B’ and ’C’ were first defined as the desired pattern. The starting point in each case was then a random distribution of 500 particles.

Broadly each shape was formed by using Bessel function shaped field cycling to first agglomerate particles to the right size and then clear out hollows where desired in the centre of the shape. The low impulse patterning is then alternated with standing wave conveyor belts to either agglomerate particles into lines or move them to the edges. The full fields can be sound in the accessible data.

## Supplementary Information

Supplementary Information 1.

Supplementary Video 1.

Supplementary Video 2.

Supplementary Video 3.

Supplementary Video 4.

Supplementary Video 5.

Supplementary Information 7.

## Data Availability

The datasets generated during and analysed during the current study are available in the Zenodo repository, 10.5281/zenodo.6379141.
