# Author Correction: Dynamic patterning of microparticles with acoustic impulse control

**DOI:** 10.1038/s41598-023-40250-1

**Published:** 2023-08-14

**Authors:** Luke Cox, Anthony Croxford, Bruce W. Drinkwater

**Affiliations:** Department of Mechanical Engineering, University of Bristol, University Walk, Bristol, BS8 1TR UK

Correction to: *Scientific Reports* 10.1038/s41598-022-18554-5, published online 25 August 2022

The Funding section in the original version of this Article was omitted. The Funding section now reads:

“The authors would like to thank the funding sources for this project, the UK Engineering and Physical Science Research Council and Ultraleap Ltd who part-funded L.C.’s CASE studentship, and the EPSRC funder Grant Number—EP/N509619/1.”

The original Article has been corrected.

