# Supplementary material for: Dynamic patterning of microparticles with acoustic impulse control
Source: Sci Rep. 2022 Aug 25;12:14549. doi: 10.1038/s41598-022-18554-5 (PMC9411184; doi:10.1038/s41598-022-18554-5)
Supplement: Supplementary file 1 — Supplementary Information 1. [file 41598_2022_18554_MOESM1_ESM.pdf]

Dynamic patterning of microparticles with acoustic impulse control Supplementary Information

Luke Cox[1,*], **Anthony Croxford**[1], **and Bruce W. Drinkwater**[1]

[1]Department of Mechanical Engineering, University of Bristol, University Walk, Bristol, BS8 1TR, UK
[*]lc13606@my.bristol.ac.uk

Comparison to Continuous Waves

In this section we demonstrate the advantage of using low impulse patterning relative to a single continuous field. As an example of a complex pattern we choose the L-shape shown in Figure 3d. To create such a pattern, the desired single continuous wave pressure field, p_{des}, is two sets of parallel lines, as shown in Supplementary Figure 1a. The inter-line distance was chosen to be equal to that between the intensity peaks of a twin trap, i.e. $\frac{2\lambda}{3}$. Other distances were tried with the results being comparable or worse. The question now is what phases and amplitudes must be generated at the array elements to achieve this desired pattern. There are a number of schemes available for solving such inverse problems and here we adopt the widely used ambisonics technique[1]. In this method a propagation matrix is formed between the sources and the desired field. The pseudoinverse, H^{pinv}, of the propagation matrix is then found via the least squares technique and used to find the optimal phase and amplitude of the sources. These source terms can then be re-propagated via equation (4) to produce the achieved pressure field and this is shown in Supplementary Figure 1b. The force field due to this optimised single continuous wave solution is shown in Supplementary Figure 1c. Note here that the force artefacts away from the desired field are now larger in magnitude than the desired trapping forces even for this relatively simple shape.

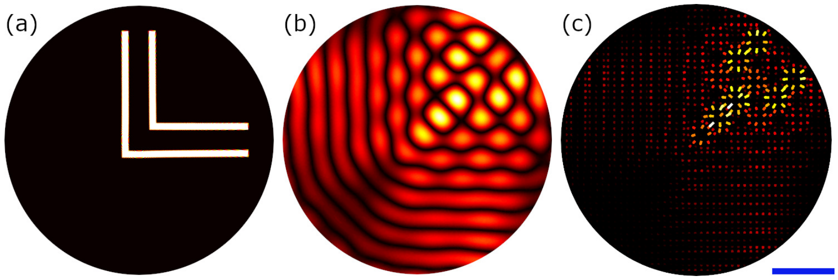

Supplementary Figure 1. A optimised single continuous wave attempt to create an L-shape via a matrix inversion approach. (a) shows the desired pressure field input to the inversion algorithm. (b) shows the output pressure field created and (c) shows the resultant force field. Note in (b & c) that the artefacts in the force field are of higher amplitude than the desired patterning forces. The blue scale bar is 1mm.

A factor against ambisonics optimisation is that it is sensitive to imperfections in the device as such imperfections mean that the true inverse problem is not solved. Focused fields and those derived from them (such as Bessel-function shapes and twin traps) are much more robust to such imperfections as they are favoured by the geometry of the device. Supplementary Figures 2 (a-c) show the force amplitudes from the summed force field shown in Figure 3c. (a) shows in the ideal simulation with identical elements. (b & c) show simulations where a random variation is applied to both the amplitude and phase of the simulated experiment. This captures the scenario when the experiment (forward model) differs from the simulation used for the inversion. In (b) this random experimental variation is limited to within ±5% whilst in (c) it is limited to ±50% of the perfect device. Supplementary Figures 2 (d-f) show the same comparison but for the ambisonics optimisation forming the field. In (d) because elements are identical the true inverse problem has been solved. This can be compared to (e & f) where the element variation leads to very poor performance. Note that whilst the breakdown in ambisonics occurs within ±5% variation, the switching approach retains good force field reproduction even at ±50% experimental variation.

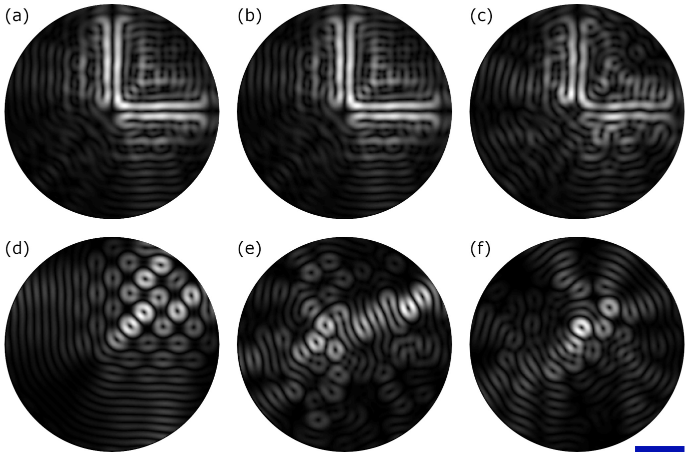

Supplementary Figure 2. A comparison of the simulated force field magnitudes for the L-shape generated by the multiplexing from Figure 3c in (a, b & c) or the single continuous wave field from Figure 1 in (d, e & f). In (a & d) the element outputs are all ideal. In (b, c, e & f) a random variation is applied to both the phase and amplitude of each of the outputs in the simulated experiment. In (b & e) the variation for both phase and amplitude is within ±5% of the original. In (c & f) it is within ±50% of the original. The blue scale bar is 1mm.

Data for simulated ABC letter patterns

The data for the discussion around Figure 8 is shown in Supplementary Table 1.

Supplementary Table 1. The average percentage of particles outside the pattern after clearing for the shapes in Figure 8. Each number is an average of 30 repetitions of the simulation from a random distribution.

Pattern	Particles Outside Pattern (%)			
	Uncleared		Cleared	
	Mean	Std Dev	Mean	Std Dev
A	60.1	2.5	0.5	0.3
B	58.1	2.6	1.2	0.4
C	81.5	1.5	1.2	0.6

Supplementary Video Information

A guide to the Supplementary Videos provided in support of this paper is shown in Supplementary Table 2.

Supplementary Table 2. A guide to the supplementary videos provided with this paper.

Supplementary Video Number	File Name	Length (mm:ss)
1	Supplementary Video 1 Tools Table	01:23
2	Supplementary Video 2 Single Line Forming	00:34
3	Supplementary Video 3 Circle Forming	00:22
4	Supplementary Video 4 Line Manipulation	01:47
5	Supplementary Video 6 Simulated ABC Forming	02:09

References

1. Kirkeby, O. & PA., N. Reproduction of plane wave sound fields. *The J. Acoust. Soc. Am.* **94**, 2992–3000, DOI: 10.1121/1.407330) (1993).
