# Supplementary material for: Dynamic patterning of microparticles with acoustic impulse control
Source: Sci Rep. 2022 Aug 25;12:14549. doi: 10.1038/s41598-022-18554-5 (PMC9411184; doi:10.1038/s41598-022-18554-5)
Supplement: Supplementary file 7 — Supplementary Information 7. [file 41598_2022_18554_MOESM7_ESM.pdf]

Supplementary Videos Information

Supplementary Video Number	File Name	Length (mm:ss)
1	Supplementary Video 1 Tools Table	01:23
2	Supplementary Video 2 Single Line Forming	00:34
3	Supplementary Video 3 Circle Forming	00:22
4	Supplementary Video 4 Line Manipulation	01:47
5	Supplementary Video 6 Simulated ABC Forming	02:09
